# Supplementary figures and images for: Fetal loss in pregnant rhesus macaques infected with high-dose African-lineage Zika virus
Source: PLoS Negl Trop Dis. 2022 Aug 4;16(8):e0010623. doi: 10.1371/journal.pntd.0010623 (PMC9380952; doi:10.1371/journal.pntd.0010623)

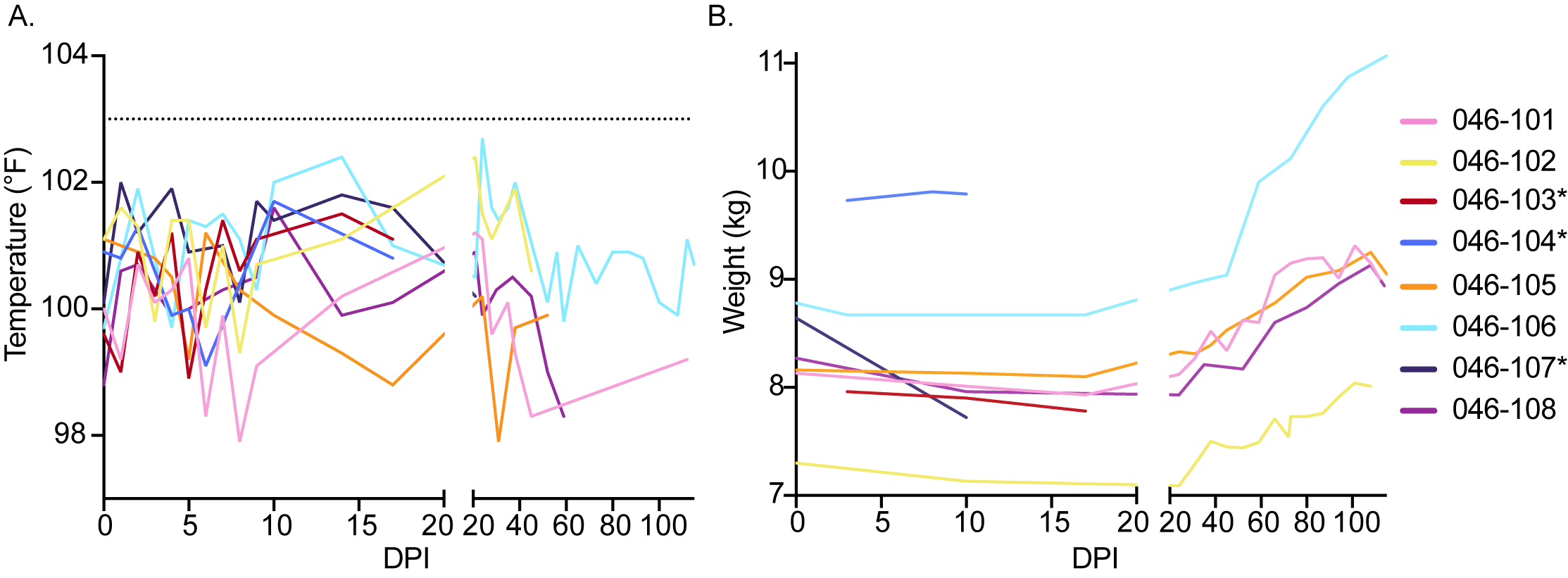

Supplement: S1 Fig — (A) Maternal temperatures over time. (B) Maternal weights over time. Dams with early fetal loss are marked with an asterisk and were not monitored after cesarean section. (TIF) [file pntd.0010623.s001.tif]

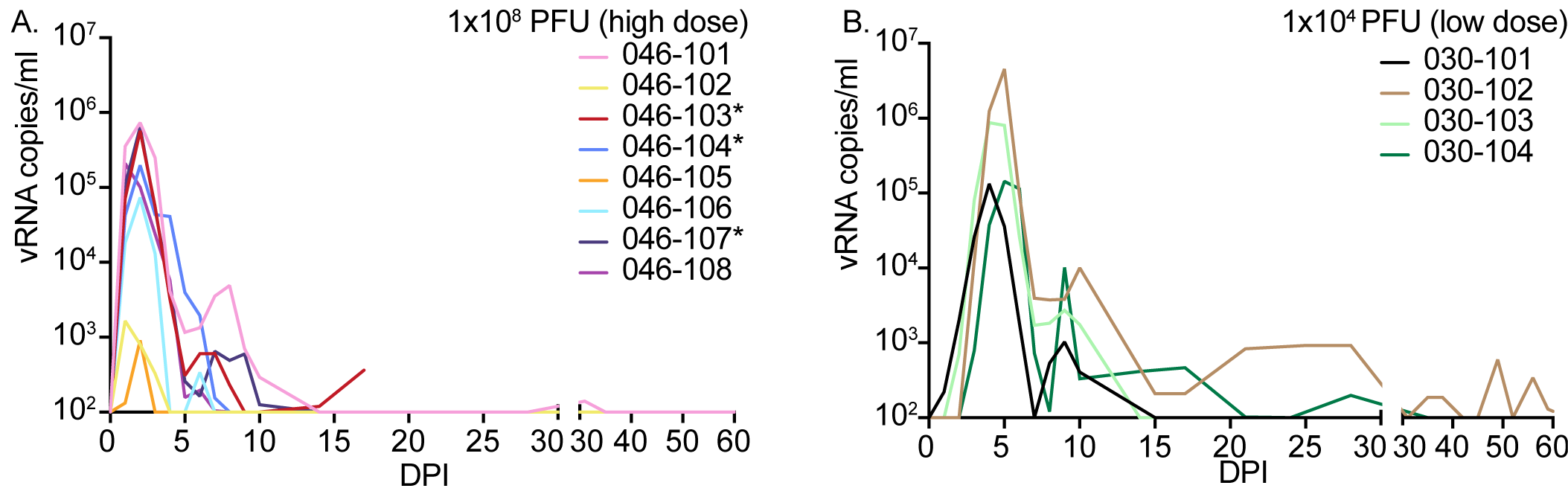

Supplement: S2 Fig — (A) Viral loads through 60 DPI in high-dose dams. Dams with early fetal loss are marked with a single asterisk. (B) Viral loads through 60 DPI in low-dose dams. (TIF) [file pntd.0010623.s002.tif]

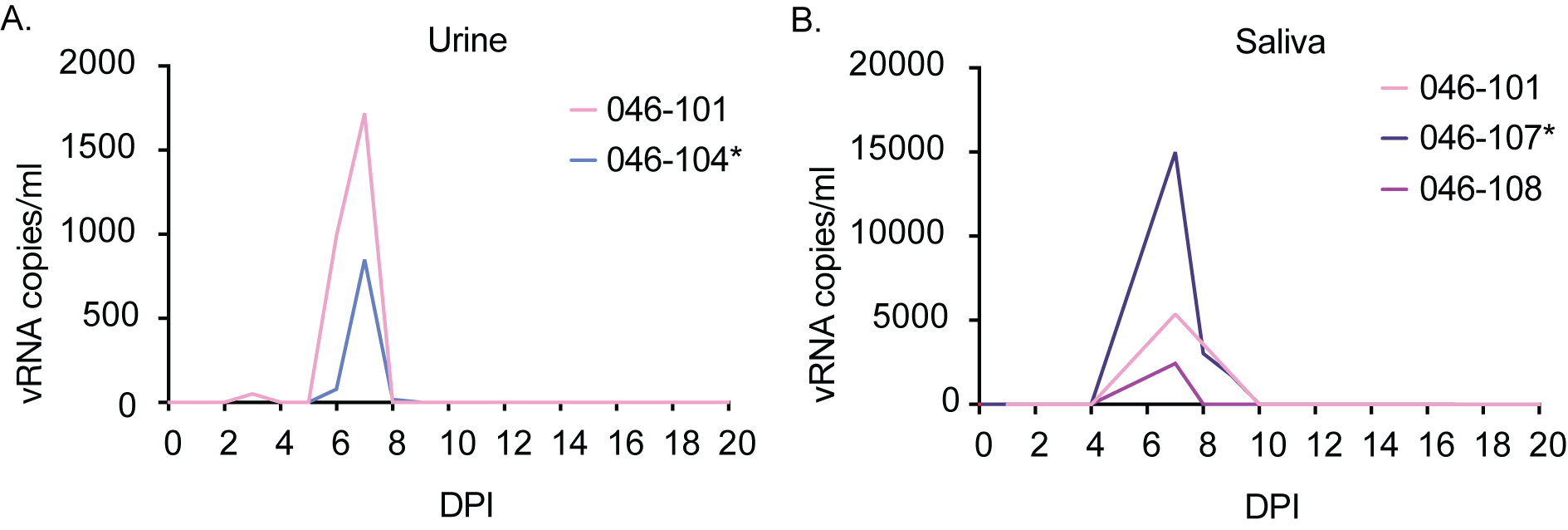

Supplement: S3 Fig — Viral loads were measured by ZIKV-specific RT-qPCR. (TIF) [file pntd.0010623.s003.tif]

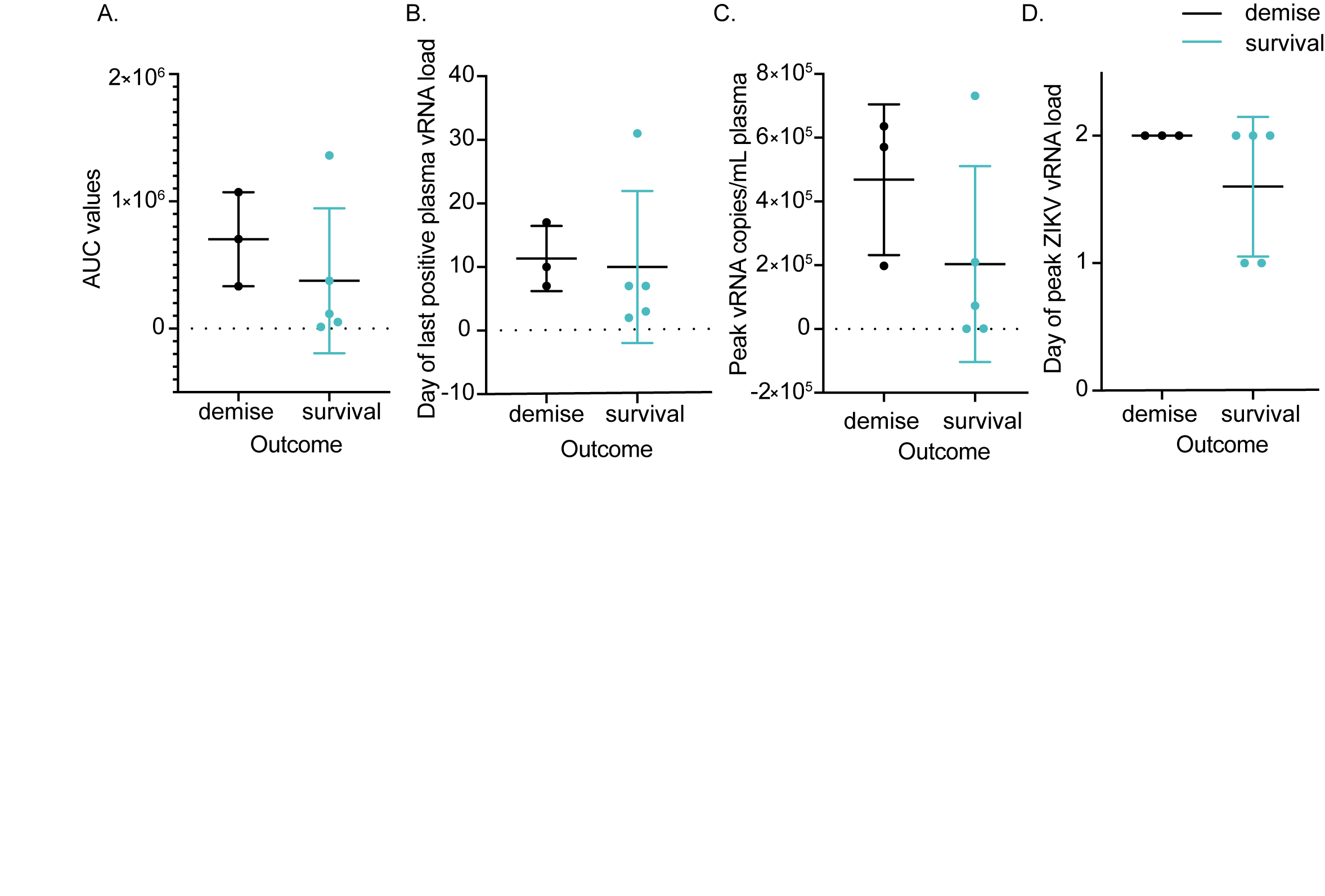

Supplement: S4 Fig — Viral loads were measured in plasma samples by ZIKV-specific qRT-PCR. (A) Comparison of area under the curve (AUC). For all graphs in parts B-E, the mean value is shown with error bars representing standard deviation. (B) Days post-infection of the last positive plasma vRNA load. (C) Peak plasma viral load in copies/ml plasma. (D) Day post-infection of peak plasma viral load. *** Represents a p-value of <0.001, while no asterisk represents no statistical difference. (TIF) [file pntd.0010623.s004.tif]

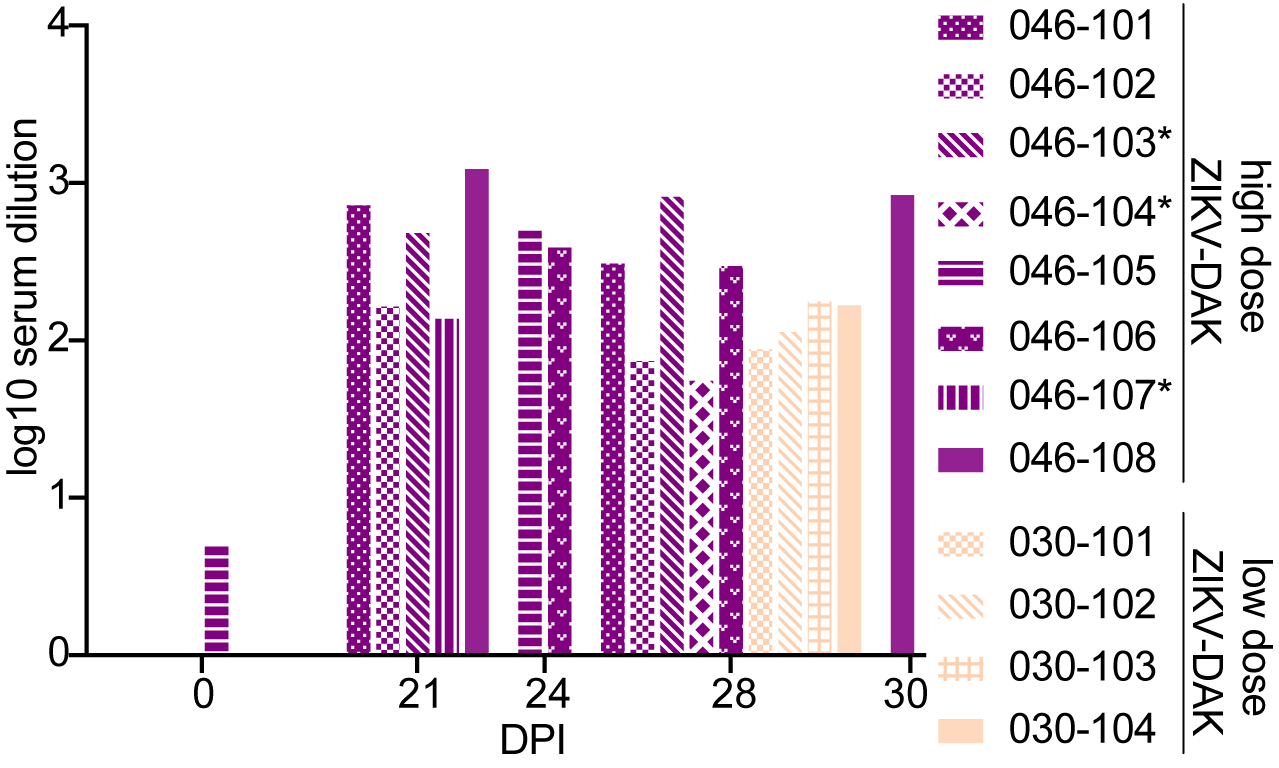

Supplement: S5 Fig — Plaque reduction neutralization tests were performed on serum samples collected 0 days post infection (DPI) and between 21 and 28 DPI from animals infected with high-dose (1x10^8^ PFU) or a low dose (104 PFU) of ZIKV-DAK. Dams with early fetal loss are marked with an asterisk. (TIF) [file pntd.0010623.s005.tif]

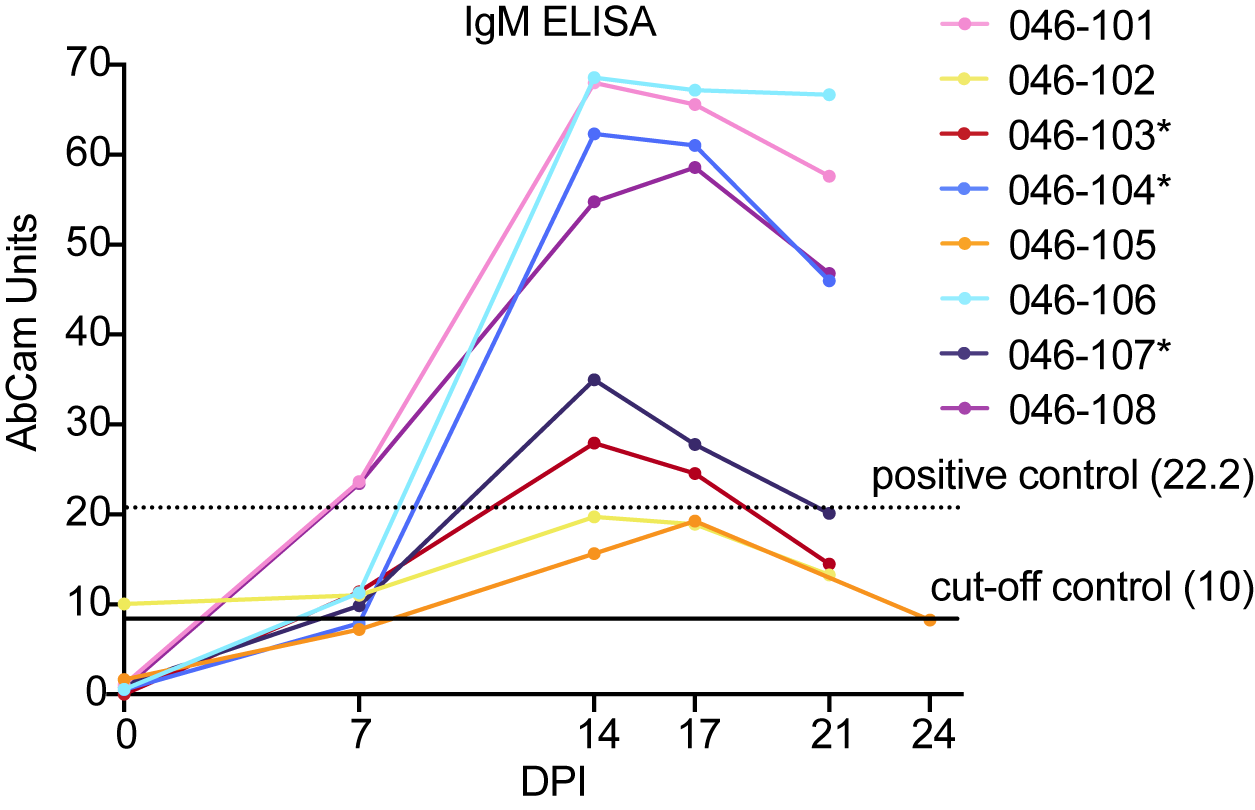

Supplement: S6 Fig — Levels of IgM antibodies were measured in maternal serum samples at 0, 7, 14, 17 and 21 or 24 DPI. Values are normalized to AbCam units for comparison based on the manufacturer’s recommendation (see materials and methods). (TIF) [file pntd.0010623.s006.tif]

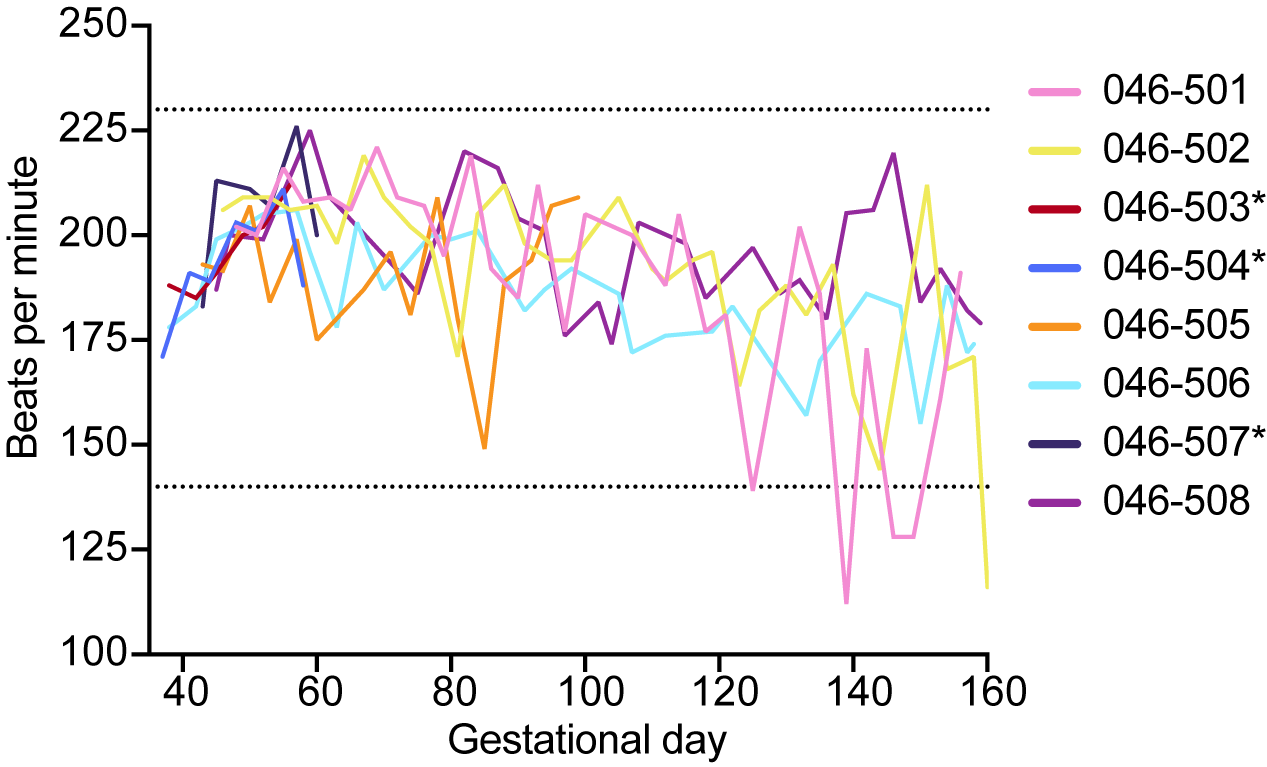

Supplement: S7 Fig — Fetal heart rate was monitored biweekly by ultrasound on high-dose (1x10^8^ PFU ZIKV-DAK) dams to confirm fetal viability. The horizontal, dotted lines on the graph represent the range of normal heart rates for fetuses at WNPRC. Cases of early fetal loss are marked with an asterisk. (TIF) [file pntd.0010623.s007.tif]

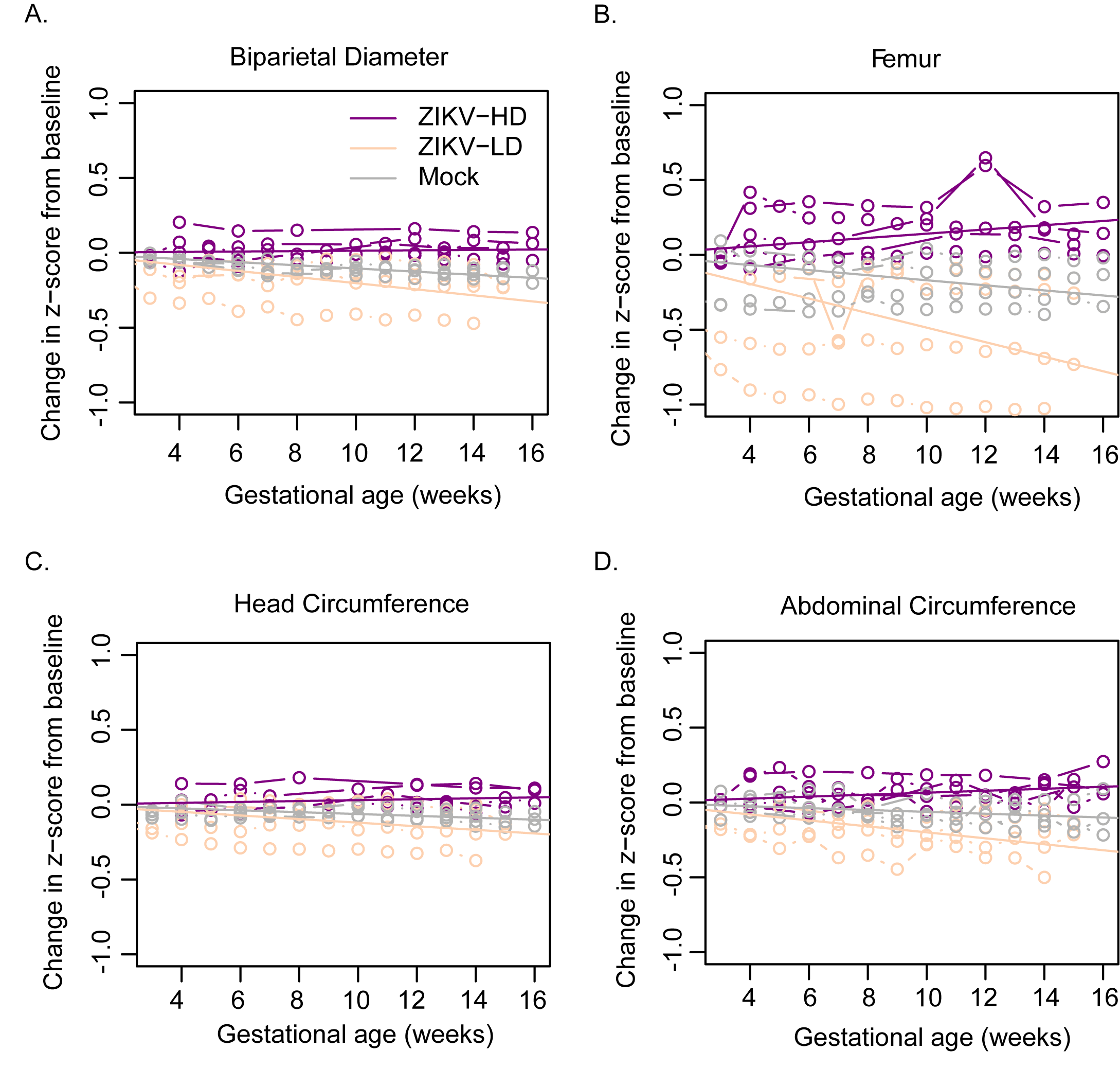

Supplement: S8 Fig — Normative data generated by Tarantal at CNPRC were used to calculate Z-scores for each animal. Open circles represent the change in Z-score from baseline for each animal. Solid lines show the growth trajectories for each group and were quantified by calculating regression slope parameters from baseline using a linear mixed-effects model with animal-specific random effects and an autoregressive correlation structure. (TIF) [file pntd.0010623.s008.tif]

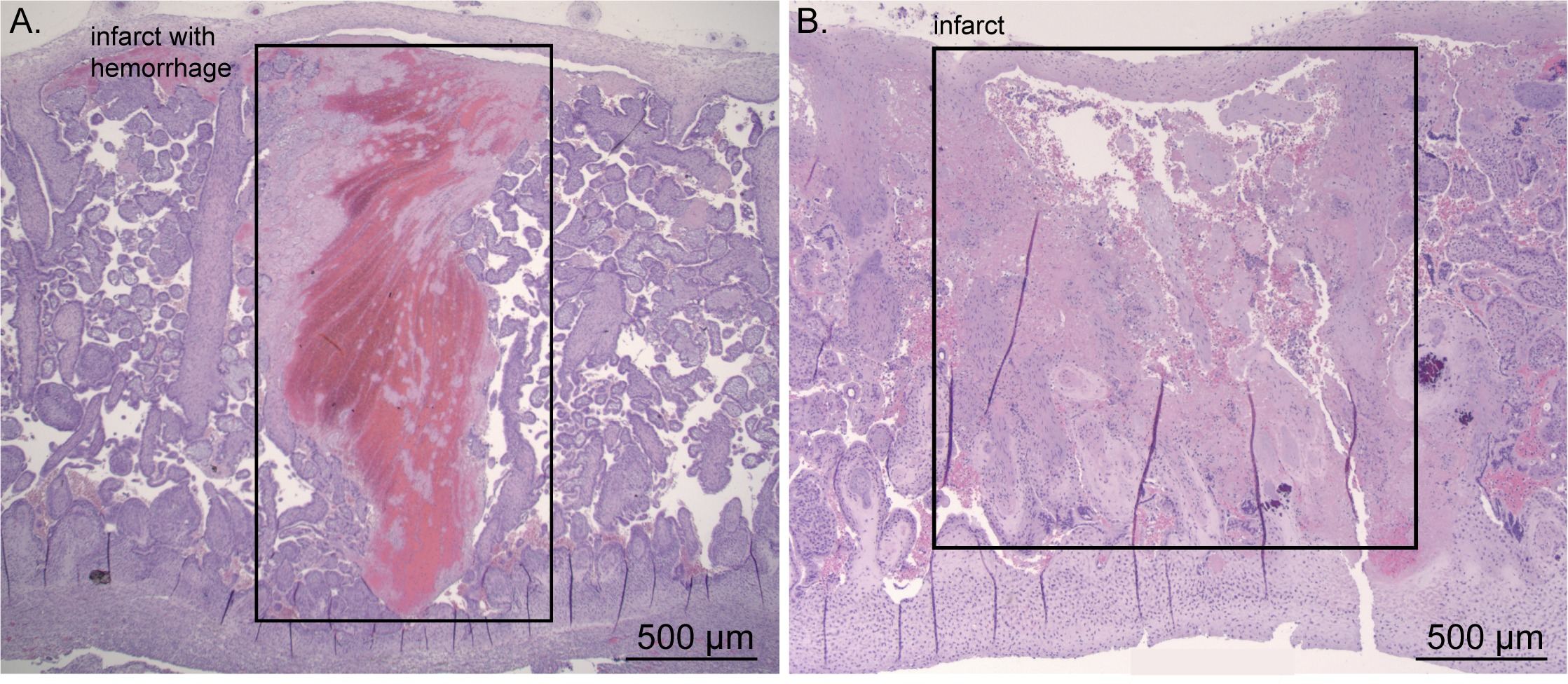

Supplement: S9 Fig — (A) Placental infarction in 046–103 and (B) 046–107. Placental tissues were stained with H&E and are imaged at 4X magnification. Black boxes denote areas of infarct. (TIF) [file pntd.0010623.s009.tif]

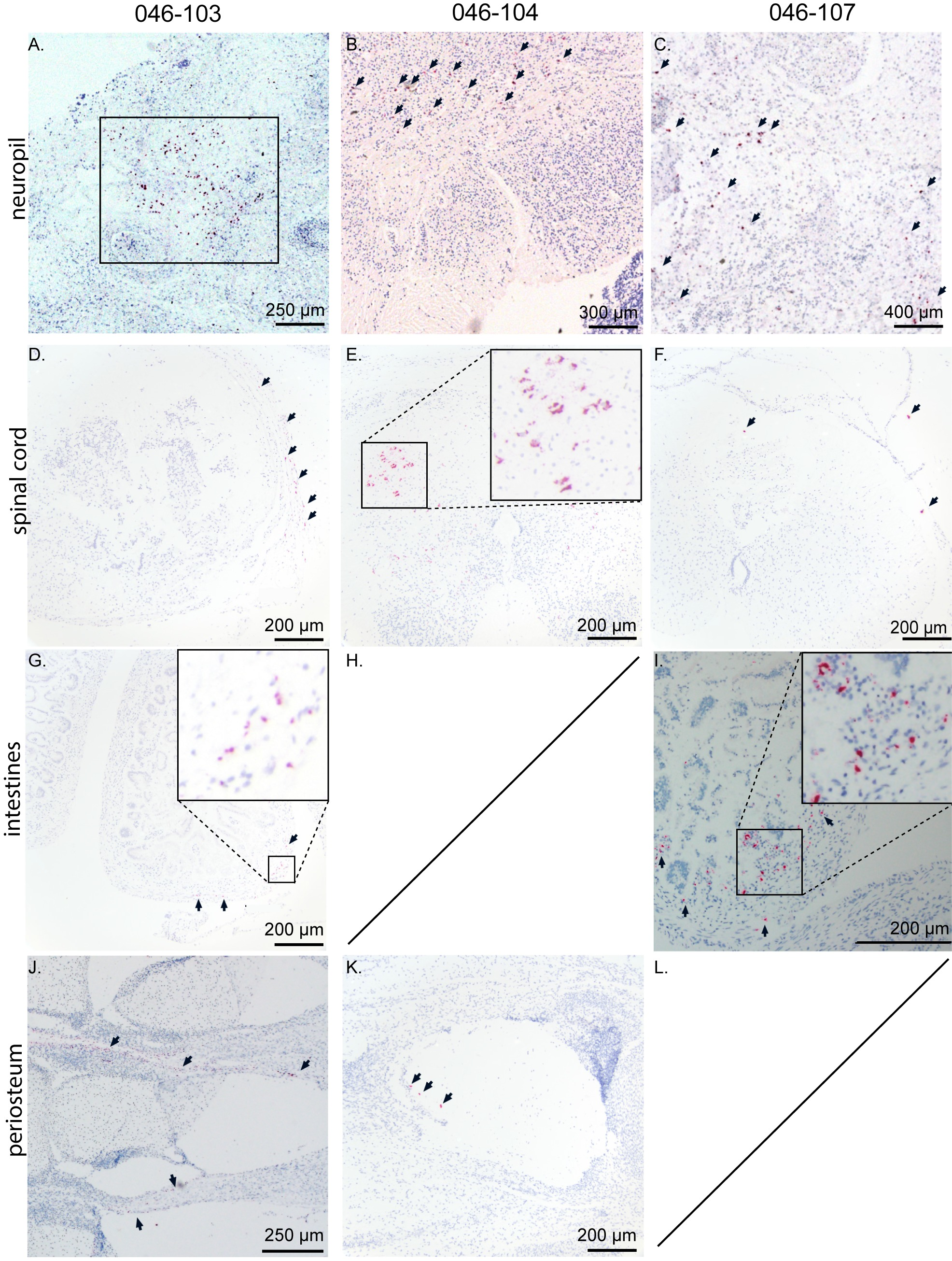

Supplement: S10 Fig — Representative images of ZIKV RNA distribution in fetal tissues from the three cases of early fetal loss (excluding the head). Foci of ZIKV RNA were detected in the neuropil of (A) 046–503 (boxed), (B) 046–504 (arrows), and (C) 046–507; the spinal cord of (D) 046–503 (arrows), (E) 046–504 (boxed), and (F) 046–507 (arrows); the intestines of (G) 046–503 (boxed, arrows) and (I) 046–507 (boxed, arrows); and the periosteum of (J) 046–503 (boxed, arrows) and (K) 046–507 (arrows). ZIKV RNA is shown in red. (TIF) [file pntd.0010623.s010.tif]

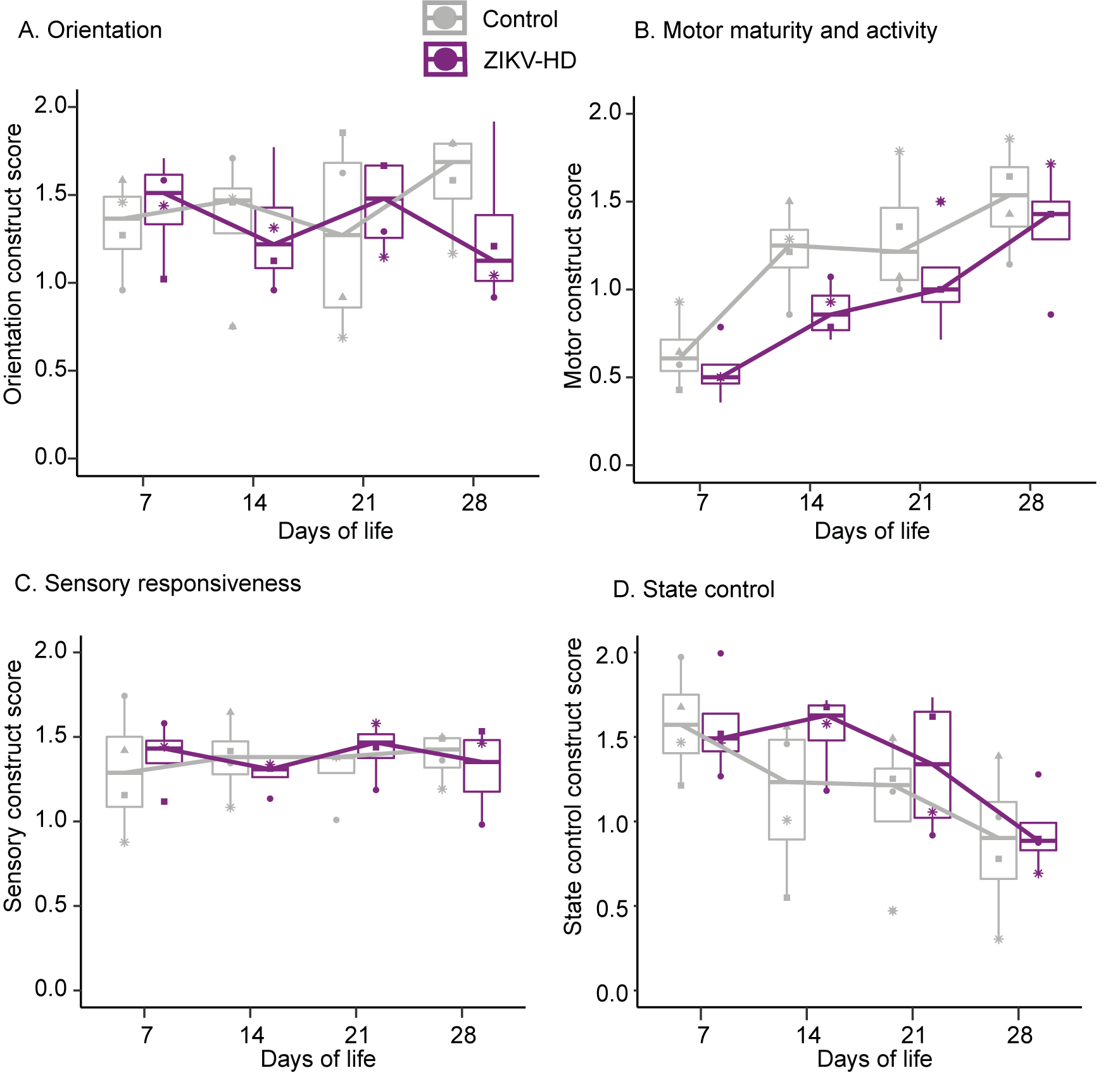

Supplement: S11 Fig — Neurodevelopment was measured by SNAP at seven, 14, 21, and 28 days of life for high-dose ZIKV-DAK exposed (ZIKV-HD) and control infants. Scores in the (A) Orientation, (B) Motor Maturity and Activity, (C) Sensory Responsiveness, and (D) State control constructs are illustrated while controlling for birth weight and days reared in nursery conditions. Animals were rated on a five point Likert scale ranging from 0 to 2 with higher scores reflecting optimal scores. For all graphs shown the results are reported in terms of model-adjusted means along with the corresponding 95% confidence intervals (95% CI). (TIF) [file pntd.0010623.s011.tif]

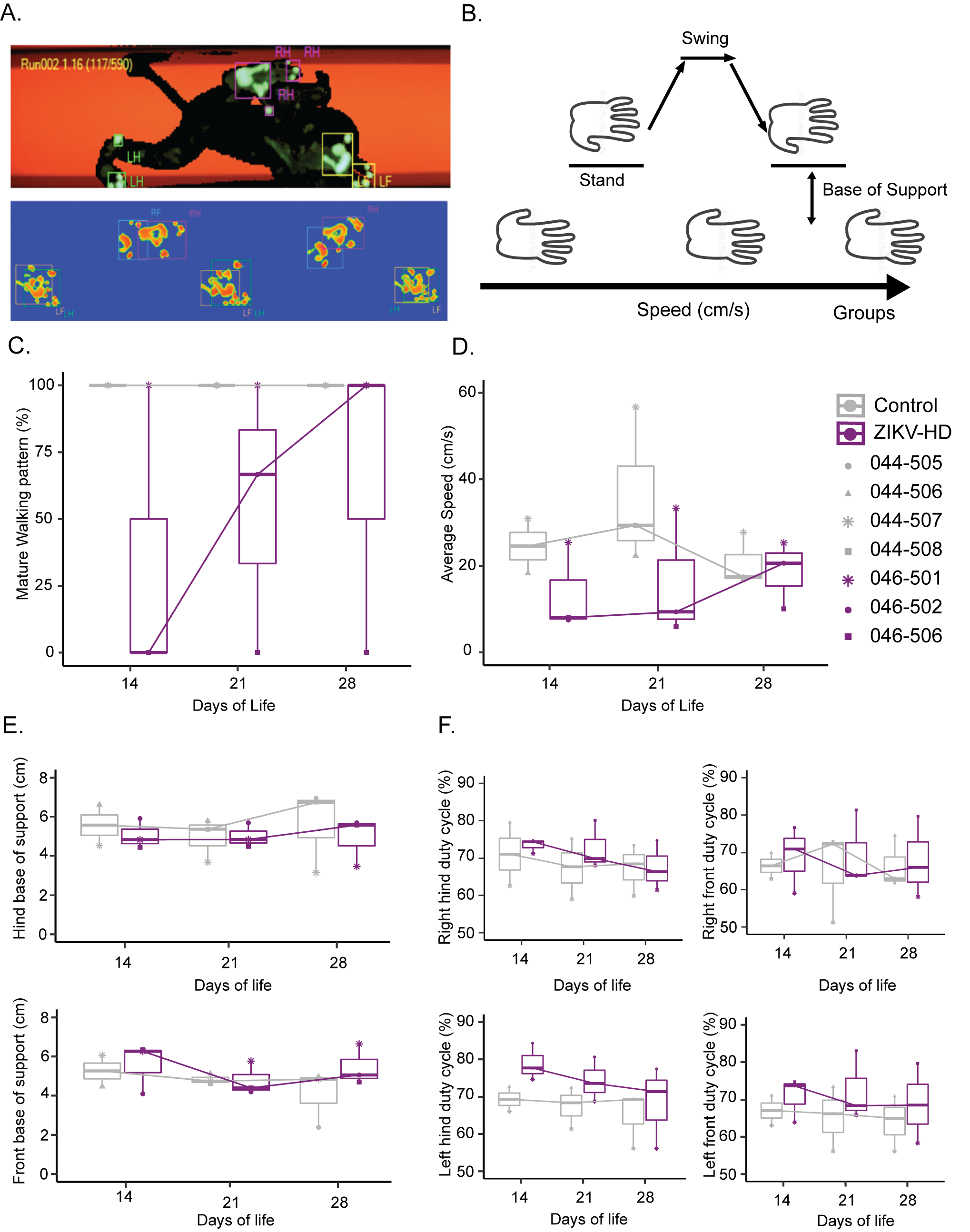

Supplement: S12 Fig — (A) Screenshot of the infant completing a run in the CatWalk Noldus XT measuring footfalls on a pressure plate that are labeled showing bilateral front (RF, LF) and hind (RH, LH) limbs. For all graphs shown in C-F, the results are reported in terms of model-adjusted means along with the corresponding 95% confidence intervals (95% CI). (B) Visual representation of gait variables including duty cycle (stand/stand + swing), base of support, and speed (cm/s), (C) % of runs in mature locomotion pattern, (D) Average speed across runs, (E) Base of support defined between the left and right foot/hand prints, and F) Duty cycle defined as the % time spent on each limb. (TIF) [file pntd.0010623.s012.tif]
