# Supplementary material for: Fetal loss in pregnant rhesus macaques infected with high-dose African-lineage Zika virus
Source: PLoS Negl Trop Dis. 2022 Aug 4;16(8):e0010623. doi: 10.1371/journal.pntd.0010623 (PMC9380952; doi:10.1371/journal.pntd.0010623)
Supplement: S1 Table — Animals were subcutaneously (SQ) exposed to either 1x10^8^ PFU ZIKV-DAK, 1x10^4^ PFU ZIKV-DAK, or PBS (mock) between GD 41 and GD 50. (DOCX) [file pntd.0010623.s013.docx]

**Table S1. Demographic characteristics of the 17 pregnant rhesus macaques on this study.** Animals were subcutaneously (SQ) exposed to either 1x10^8^ PFU ZIKV-DAK, 1x10^4^ PFU ZIKV-DAK, or PBS between GD 41 and GD 50.

| **Dam ID** | **Infant/Fetal ID** | **Target Virus Exposure Dose** | **GD of Infection** | **Baseline weight (kg)** | **Age (yr)** | **GD/DPI of fetal loss** | **GD of C-section** |
| --- | --- | --- | --- | --- | --- | --- | --- |
| 046-101 | 046-501 | 1x10^8^ PFU | 48 | 8.13 | 14.2 | - | 160 |
| 046-102 | 046-502 | 1x10^8^ PFU | 46 | 7.13 | 14 | - | 160 |
| 046-103 | 046-503 | 1x10^8^ PFU | 42 | 7.81 | 14.6 | 59/17 | - |
| 046-104 | 046-504 | 1x10^8^ PFU | 41 | 9.73 | 16.6 | 62/21 | - |
| 046-105 | 046-505 | 1x10^8^ PFU | 43 | 8.16 | 16.5 | - | 159 |
| 046-106 | 046-506 | 1x10^8^ PFU | 42 | 8.78 | 8.2 | - | 158 |
| 046-107 | 046-507 | 1x10^8^ PFU | 43 | 8.64 | 12.2 | 64/21 | - |
| 046-108 | 046-508 | 1x10^8^ PFU | 45 | 8.27 | 11.5 | - | 159 |
| 030-101 | 030-501 | 1x10^4^ PFU | 48 | 8.14 | 10.8 | - | 154 |
| 030-102 | 030-502 | 1x10^4^ PFU | 44 | 8.56 | 19.0 | - | 156 |
| 030-103 | 030-503 | 1x10^4^ PFU | 46 | 9.92 | 13.4 | - | 159 |
| 030-104 | 030-504 | 1x10^4^ PFU | 50 | 8.31 | 15.2 | - | 155 |
| 020-101 | 020-501 | PBS | 45 | 6.83 | 11.5 | - | 158 |
| 044-105 | 044-505 | PBS | 42 | 6.06 | 11.4 | - | 161 |
| 044-106 | 044-506 | PBS | 48 | 9.36 | 15.4 | - | 160 |
| 044-107 | 044-507 | PBS | 48 | 8.00 | 8.5 | - | 163 |
