# Supplementary material for: Fetal loss in pregnant rhesus macaques infected with high-dose African-lineage Zika virus
Source: PLoS Negl Trop Dis. 2022 Aug 4;16(8):e0010623. doi: 10.1371/journal.pntd.0010623 (PMC9380952; doi:10.1371/journal.pntd.0010623)
Supplement: S2 Table — (DOCX) [file pntd.0010623.s014.docx]

**Table S2. In-utero imaging observations and interpretations from the dams that received high-dose ZIKV-DAK.**

| Animal ID | Ultrasound Interpretations |
| --- | --- |
| 046-101 | Ultrasound remarks: Slightly bright bowel noticed on GD 55, amnion chorion not fused on GD 66. Edge of placenta raised, similar to circumvallate placenta on GD 100. Started to breach on GD 156, 4 days before delivery.  Placenta notes: calcifications in the decidua on GD 65, increased on GD 121 and dense calcifications maintained until birth. |
| 046-102 | Ultrasound remarks: Amnion chorion not fused as of GD 46 . On GD 63 the chest wall is thick, similar to skin edema. On GD 70 cardiac to chest ratio is smaller than expected but is back to normal on GD 91.  Placenta notes: Calcifications on the decidual border noted on GD 77. Calcifications noted throughout placenta on GD 112. Dense placental calcifications noted on GD 147, these increase in density up until GD 154. |
| 046-103* | Ultrasound remarks: Falx, thalamus, and choroid plexus seen GD 52. Amnion chorion not fused up until day of demise, on GD 59. Skin is also thick and edematous.  Placenta notes: No calcifications seen throughout. |
| 046-104* | Ultrasound remarks: Slightly full nuchal area on GD 55, unknown significance in rhesus, may be an early indicator of edema/hydrops. On GD 58 the amnion and chorion were still not fused and a small amount of fluid was noticed around the heart. The fluid did not extend past the AV node so it was not concerning. Heart beat no longer detected on GD 62, skin was edematous.  Placenta notes: No calcifications seen. |
| 046-105 | Ultrasound remarks: placenta previa over the cervix on GD 39. Cardiac to chest ratios appear small on GD 67 but are back to normal on GD 81.  Placenta notes: rare calcifications along decidual edge noted on GD 67, increase in overall calcifications on GD 109 and GD 137. |
| 046-106 | Ultrasound remarks: skin appears slightly thick on GD 45. AEDF** noted on GD 73 and GD 87.  Placenta notes: scant calcifications noted on GD 73, increasing in density throughout the placenta but notably on the decidual edge up until delivery. |
| 046-107* | Ultrasound remarks: Amnion chorion not fused. Absent end diastolic flow on GD 53. Heart beat no longer detected on GD 64, body wall was edematous.  Placenta notes: No noted calcifications. |
| 046-108 | Ultrasound remarks: on GD 45 there was some skin edema that is not quite hydrops because fluid is not in any other compartments. AEDF** on GD 55, GD 69, and GD 80. Echogenic foci near the midline of the right ventricle on GD 69 this is considered to be a soft marker for trisomy 21. On GD 69 the esophagus appears to be moving upward through the diaphragm (hiatal hernia).  Placenta notes: scant placental calcifications starting on GD 80. Increased calcifications on decidual edge GD 97. |

*Fetal demise

**Absent End-Diastolic Flow
