# Supplementary material for: Fetal loss in pregnant rhesus macaques infected with high-dose African-lineage Zika virus
Source: PLoS Negl Trop Dis. 2022 Aug 4;16(8):e0010623. doi: 10.1371/journal.pntd.0010623 (PMC9380952; doi:10.1371/journal.pntd.0010623)
Supplement: S3 Table — Z-scores are log-transformed ratios. Animals in high-dose ZIKV-DAK, low-dose ZIKV-DAK, and mock groups were compared. (DOCX) [file pntd.0010623.s015.docx]

Table S3. Slope parameters for changes in z-scores for in-utero measurements across gestation, stratified by group. Z-scores are log-transformed ratios. Animals in high-dose ZIKV-DAK, low-dose ZIKV-DAK, and mock groups were compared.

| Outcome | Group | Slope | 95% CI of Slope | p-value^1^ |
| --- | --- | --- | --- | --- |
| BPD-z-score | 1:ZIKV_HD | 0.001 | -0.005-0.008 | 0.6731 |
| BPD-z-score | 2:ZIKV_LD | -0.014 | -0.023--0.005 | 0.0020 |
| BPD-z-score | 3:Mock | -0.009 | -0.017--0.001 | 0.0322 |
| Femur-z-score | 1:ZIKV_HD | 0.010 | -0.008-0.028 | 0.2796 |
| Femur-z-score | 2:ZIKV_LD | -0.016 | -0.042-0.009 | 0.1992 |
| Femur-z-score | 3:Mock | -0.005 | -0.027-0.017 | 0.6680 |
| Abdominal-z-score | 1:ZIKV_HD | 0.008 | -0.002-0.018 | 0.1214 |
| Abdominal-z-score | 2:ZIKV_LD | -0.010 | -0.022-0.003 | 0.1248 |
| Abdominal-z-score | 3:Mock | -0.005 | -0.015-0.006 | 0.3781 |
| Head-Circ-z-score | 1:ZIKV_HD | 0.001 | -0.004-0.007 | 0.6454 |
| Head-Circ-z-score | 2:ZIKV_LD | -0.010 | -0.018--0.002 | 0.0105 |
| Head-Circ-z-score | 3:Mock | -0.004 | -0.011-0.003 | 0.2820 |
| Head-Circ/Femur-Ratio | 1:ZIKV_HD | -0.052 | -0.073--0.031 | <0.0001 |
| Head-Circ/Femur-Ratio | 2:ZIKV_LD | -0.052 | -0.08--0.024 | 0.0004 |
| Head-Circ/Femur-Ratio | 3:Mock | -0.061 | -0.086--0.037 | <0.0001 |
| Biparietal-Diameter/Femur-Ratio | 1:ZIKV_HD | -0.059 | -0.078--0.039 | <0.0001 |
| Biparietal-Diameter/Femur-Ratio | 2:ZIKV_LD | -0.056 | -0.083--0.029 | <0.0001 |
| Biparietal-Diameter/Femur-Ratio | 3:Mock | -0.067 | -0.09--0.043 | <0.0001 |

^1^p-value for testing whether slope is different from 0
