# Supplementary material for: Fetal loss in pregnant rhesus macaques infected with high-dose African-lineage Zika virus
Source: PLoS Negl Trop Dis. 2022 Aug 4;16(8):e0010623. doi: 10.1371/journal.pntd.0010623 (PMC9380952; doi:10.1371/journal.pntd.0010623)
Supplement: S4 Table — Animals in high-dose ZIKV-DAK, low-dose ZIKV-DAK, and mock groups were compared. (DOCX) [file pntd.0010623.s016.docx]

Table S4. Pairwise comparisons between groups of slopes for z-scores of in-utero measurements. Animals in high-dose ZIKV-DAK, low-dose ZIKV-DAK, and mock groups were compared.

| Outcome | Comparison | p-value |
| --- | --- | --- |
| BPD_z-score | ZIKV-HD vs ZIKV-LD | 0.0062 |
| BPD_z-score | ZIKV-HD vs Mock | 0.0545 |
| BPD_z-score | ZIKV-LD vs Mock | 0.3749 |
| Femur_z-score | ZIKV-HD vs ZIKV-LD | 0.0950 |
| Femur_z-score | ZIKV-HD vs Mock | 0.3062 |
| Femur_z-score | ZIKV-LD vs Mock | 0.4896 |
| Abdominal_z-score | ZIKV-HD vs ZIKV-LD | 0.0312 |
| Abdominal_z-score | ZIKV-HD vs Mock | 0.0895 |
| Abdominal_z-score | ZIKV-LD vs Mock | 0.5508 |
| Head_Circ_z-score | ZIKV-HD vs ZIKV-LD | 0.0189 |
| Head_Circ_z-score | ZIKV-HD vs Mock | 0.2621 |
| Head_Circ_z-score | ZIKV-LD vs Mock | 0.2137 |
| Head_Circ/Femur_Ratio | ZIKV-HD vs ZIKV-LD | 0.9900 |
| Head_Circ/Femur_Ratio | ZIKV-HD vs Mock | 0.5701 |
| Head_Circ/Femur_Ratio | ZIKV-LD vs Mock | 0.6335 |
| Biparietal_Diameter/Femur_Ratio | ZIKV-HD vs ZIKV-LD | 0.8776 |
| Biparietal_Diameter/Femur_Ratio | ZIKV-HD vs Mock | 0.6001 |
| Biparietal_Diameter/Femur_Ratio | ZIKV-LD vs Mock | 0.5538 |
