# Supplementary material for: Fetal loss in pregnant rhesus macaques infected with high-dose African-lineage Zika virus
Source: PLoS Negl Trop Dis. 2022 Aug 4;16(8):e0010623. doi: 10.1371/journal.pntd.0010623 (PMC9380952; doi:10.1371/journal.pntd.0010623)
Supplement: S5 Table — Analysis was performed between infants in high-dose ZIKV-DAK and mock groups. Because gender was confounded with group it was not included as a covariate in this analysis. (DOCX) [file pntd.0010623.s017.docx]

Table S5. Statistical analyses comparing weights, head circumference (HC), biparietal diameter (BPD), and weight. Analysis was performed between infants in high-dose ZIKV-DAK and mock groups. Because gender was confounded with group it was not included as a covariate in this analysis

|  | Mock  N=5 | | HD  N=5 | | LD  N=4 | |  |  |  |  |
| --- | --- | --- | --- | --- | --- | --- | --- | --- | --- | --- |
|  | Mean^?^ | 95% CI | Mean^?^ | 95% CI | Mean^?^ | 95% CI | p-value | p-value^1^ | p-value^2^ | p-value^3^ |
| Weight (gram) | 507 | 439-575 | 544 | 498-589 | 466 | 392-540 | 0.1334 | 0.3087 | 0.4565 | 0.0849 |
| HC (mm) | 198 | 190-207 | 196 | 190-202 | 193 | 183-202 | 0.7116 | 0.5777 | 0.4260 | 0.5437 |
| BPD (mm) | 51 | 48-54 | 52 | 50-54 | 50 | 47-53 | 0.3495 | 0.4081 | 0.7237 | 0.2506 |

^?^adjusted by gestational day, dam’s age and weight

^1^p-value of comparison Mock vs. HD groups

^2^p-value of comparison Mock vs. LD groups

^3^p-value of comparison HD vs. LD groups
