# Supplementary material for: Fetal loss in pregnant rhesus macaques infected with high-dose African-lineage Zika virus
Source: PLoS Negl Trop Dis. 2022 Aug 4;16(8):e0010623. doi: 10.1371/journal.pntd.0010623 (PMC9380952; doi:10.1371/journal.pntd.0010623)
Supplement: S6 Table — Comparisons of APGAR scores were made between infants in high-dose, low-dose, and mock groups. Because gender was confounded with group it was not included as a covariate in this analysis. (DOCX) [file pntd.0010623.s018.docx]

Table S6. Statistical analyses comparing APGAR scores at one, five, and 10 minutes of life. Comparisons of APGAR scores were made between infants in high-dose, low-dose, and mock groups. Because gender was confounded with group it was not included as a covariate in this analysis.

|  | Mock  N=5 | | HD  N=5 | |  |
| --- | --- | --- | --- | --- | --- |
| APGAR | **Adjusted Mean*** | **95% CI** | **Adjusted Mean^?^** | **95% CI** | **p-value** |
| 1 min | 6.5 | 4.2-8.8 | 5.0 | 2.7-7.3 | 0.3385 |
| 5 min | 7.7 | 6.1-9.3 | 8.7 | 7.3-10.2 | 0.2883 |
| 10 min | 8.9 | 7.9-10.0 | 8.6 | 7.6-9.5 | 0.5549 |

*adjusted by gestational day, dam’s age and weight
