# Supplementary material for: Fetal loss in pregnant rhesus macaques infected with high-dose African-lineage Zika virus
Source: PLoS Negl Trop Dis. 2022 Aug 4;16(8):e0010623. doi: 10.1371/journal.pntd.0010623 (PMC9380952; doi:10.1371/journal.pntd.0010623)
Supplement: S7 Table — Characteristics of gestational day (GD), dam weight, dam age, and fetal gender were compared between mock (n = 4), high-dose (n = 5), and low-dose (n = 4) groups. (DOCX) [file pntd.0010623.s019.docx]

Table S7. Statistical analysis comparing at-birth demographic characteristics. Characteristics of gestational day (GD), dam weight, dam age, and fetal gender were compared between mock (n=4), high-dose (n=5), and low-dose (n=4) groups.

|  | Mock  N=5 | | HD  N=5 | | LD  N=4 | |  |  |  |  |
| --- | --- | --- | --- | --- | --- | --- | --- | --- | --- | --- |
|  | Mean | SD | Mean | SD | Mean | SD | p-value | p-value^1^ | p-value^2^ | p-value^3^ |
| GD | 160.6 | 2.4 | 159.2 | 0.8 | 156.0 | 2.2 | 0.0128 | 0.2703 | 0.0042 | 0.0294 |
| Dam weight | 8.4 | 1.5 | 9.2 | 1.1 | 9.8 | 0.9 | 0.2925 | 0.3324 | 0.1277 | 0.4809 |
| Dam age | 12.0 | 2.5 | 12.0 | 3.1 | 14.3 | 3.8 | 0.5149 | 0.9999 | 0.3351 | 0.3109 |
|  | N | % | N | % | N | % |  |  |  |  |
| Gender  Female  Male | 5  0 | 100  0 | 3  2 | 60  40 | 4  0 | 100  0 | 0.2857 | 0.4444 | 0.9999 | 0.444 |

^1^p-value of comparison Mock vs. HD groups

^2^p-value of comparison Mock vs. LD groups

^3^p-value of comparison HD vs. LD groups
