# Supplementary material for: Fetal loss in pregnant rhesus macaques infected with high-dose African-lineage Zika virus
Source: PLoS Negl Trop Dis. 2022 Aug 4;16(8):e0010623. doi: 10.1371/journal.pntd.0010623 (PMC9380952; doi:10.1371/journal.pntd.0010623)
Supplement: S8 Table — (DOCX) [file pntd.0010623.s020.docx]

Table S8. Morphological diagnoses of MFI and fetal tissue from animals in high-dose, low-dose, and control groups.

| Dose | ID | GA | Morphological diagnoses |
| --- | --- | --- | --- |
| 1x10^8^ PFU  ZIKV-DAK | 046-101 | 160 | Placenta primary disc: placenta, trophoblastic shell and villi have focal extensive transmural coagulative necrosis (acute to subacute) involving approximately 20% of the trophoblastic shell-villi section. In the decidua basalis, there is random and perivascular lymphoplasmacytic deciduitis (mild-moderate).  Fetal membranes and decidua parietalis: multifocal decidual vascular fibrinoid necrosis (mild-moderate). Multifocal decidual necrosis and neutrophilic infiltration (mild-moderate). |
| 1x10^8^ PFU  ZIKV-DAK | 046-102 | 160 | Placenta primary disc: multifocal decidual lymphoplasmacytic infiltration (mild). Multifocal coagulative necrosis rimmed by syncytial cell aggregates at the trophoblastic shell-villi interface (mild-moderate). Focal extensive transmural coagulative necrosis (moderate-severe) in the villi area rimmed by syncytial knot formations and mildly infiltrated by neutrophils.  Placenta secondary disc: Focal extensive transmural trophoblastic-villi to subchorionic coagulative infarction (acute-subacute). In the villi and subchorionic space, there is focal extensive remote infarction, moderately infiltrated by neutrophils and rimmed by multifocal syncytial cell knot formations. In the villi there is also transmural coagulative necrosis rimmed by syncytial cell knot formations.  Fetal membranes: Necrosis of the decidual parietalis (mild-multifocal) with neutrophil infiltration (mild-multifocal). |
| 1x10^8^ PFU  ZIKV-DAK | 046-103 | 59 | Placenta primary disc: focal necrosis of the trophoblastic shell-anchoring villi interface (acute) with hemorrhage and fibrin deposition. Mild neutrophilic infiltration.  Secondary disc: In the trophoblastic shell-subchorionic area there is focal transmural hemorrhagic-coagulative necrosis (extensive). In the intervillary space, there is multifocal fibrinoid deposition (mild).  Umbilical cord: Moderate multifocal neutrophilic umbilical vasculitis (phlebitis and arteritis) and perivasculitis. Funisitis (mild-moderate). Postmortem tissue autolysis (moderate).  Decidua: Multifocal hemosiderosis and siderophagocytosis (mild-moderate). Multifocal interstitial random and perivascular lymphocytic infiltration (mild-moderate).  Maternal liver: Multifocal portal and periportal lymphoplasmacytic and eosinophilic hepatitis with bile duct hyperplasia and reduplication (mild).  Maternal spleen: follicular lymphoid hyperplasia (moderate).  Fetus: postmortem tissue autolysis (moderate-advanced) |
| 1x10^8^ PFU  ZIKV-DAK | 046-104 | 62 | Placenta: chorionic edema, multifocal villous stromal vascular karyorrhexis (minimal). Multifocal decidual necrosis and decidual hemorrhage (mild).  Fetus: Autolysis with dermal edema (mild-moderate). |
| 1x10^8^ PFU  ZIKV-DAK | 046-105 | 159 | Placenta secondary disc: Multifocal decidual and trophoblastic shell coagulative necrosis (moderate) with intralesional neutrophilic infiltration (moderate). Multifocal hemorrhage (acute-subacute). |
| 1x10^8^ PFU  ZIKV-DAK | 046-106 | 158 | Placenta primary disc: Transmural villous necrosis (moderate, subacute) with a focal area of histiocytic infiltration. Minimal numbers of lymphocytes and plasma cells. Multifocal intralesional areas of hemorrhage and fibrin deposition. Well delimited by syncytiotrophoblast knots.  Placenta secondary disc, mid to peripheral section, decidua basalis: multifocal areas of coagulative necrosis with occasional intralesional vascular fibrin thrombi and moderate infiltration by non-degenerate neutrophils.  Decidua parietalis: Focal coagulative necrosis (moderate-extensive) with neutrophilic infiltration (moderate). Multifocal intralesional blood vessels with fibrin thrombosis. |
| 1x10^8^ PFU  ZIKV-DAK | 046-107 | 64 | Placenta: coagulative to lytic necrosis (mild-multifocal) with focal hemorrhage (acute) and hemosiderin.  Umbilical cord: edema and fibronecrotic vasculitis with lymphocytes and neutrophils (mild).  Fetal membranes: Multifocal hemorrhage (acute, mild)  Uterus: Multifocal perivascular lymphocytic myometritis with acute hemorrhage (mild). |
| 1x10^8^ PFU  ZIKV-DAK | 046-108 | 159 | Decidua parietalis: Scattered hemosiderin and mural hypertrophy of decidual vessels (mild). Multifocal intraluminal fibrin thrombi (mild).  Decidua basalis: chronic deciduitis and persistently muscularized decidual arterioles (mild). Multifocal intravascular thrombi. Multifocal hemosiderin accumulation (minimal-mild)  Placenta: Multifocal intervillositis (acute, minimal). Multifocal mineralization and multifocal transmural infarction.  Infant (046-508) necropsy report: The baby (5-day old infant male) was severely dehydrated and had no milk in the stomach at the time of necropsy. The placenta cultures returned negative for bacterial growth; however, cultures of the lung and liver were positive for mixed bacteria. Waterhouse-Friderichsen syndrome (adrenal hemorrhage) is often associated with stress, and/or sepsis. However, it is not a pathognomonic lesion for any particular disease. Presence of inflammation in multiple organs suggests systemic infection. No external wounds are observed on the infant. |
| 1x10^4^ PFU  ZIKV-DAK | 030-101 | 153 | Placenta primary disc: Multifocal neutrophilic subchorionitis (acute, minimal). Multifocal villitis (subacute, mild). Transmural villous ischemia. Multifocal basal plate infarction (mild) and multifocal mineralization (mild).  Placenta secondary disc: Multifocal villitis (chronic, moderate) and multifocal mineralization (mild).  Fetal inguinal lymph node: diffuse neutrophilic lymphadenitis (acute, moderate).  Fetal mesenteric lymph node: multifocal neutrophilic lymphadenitis (minimal).  Fetal tracheobronchial lymph node: diffuse neutrophilic lymphadenitis (minimal).  Maternal spleen: diffuse neutrophilic splenitis (minimal-mild).  Maternal liver: Multifocal centrilobular and periportal neutrophilic and lymphocytic hepatitis (mild).  Fetal ear (left): Hemorrhage with neutrophilic otitis media (minimal). |
| 1x10^4^ PFU  ZIKV-DAK | 030-102 | 156 | Placenta: multifocal transmural infarction (moderate) with acute inflammation.  Decidua basalis: Multifocal neutrophilic deciduitis (minimal-mild), multifocal hemosiderin (mild), and rare persistently muscularized decidual arteries.  Decidua parietalis: multifocal neutrophilic deciduitis (moderate), persistent muscularized decidual arteries, multifocal hemosiderin accumulation (mild).  Fetal pancreatic lymph node, submandibular lymph node, and tracheobronchial lymph node: multifocal neutrophilic lymphadenitis (mild).  Fetal axillary lymph node: neutrophilic lymphadenitis (minimal). |
| 1x10^4^ PFU  ZIKV-DAK | 030-103 | 159 | Fetal membranes: decidua parietalis has multifocal, arcuate, vascular muscularization (moderate).  Maternal spleen: splenic-follicular, lymphoid hyperplasia (moderate).  Placenta primary disc: focal extensive transmural placenta-villi coagulative to lytic necrosis with intralesional basal plate thromboses (moderate). Decidual, multifocal, coagulative necrosis (mild-moderate). Decidua basal plate has multifocal coagulative necrosis (moderate) and multifocal vascular muscularization (moderate). |
| 1x10^4^ PFU  ZIKV-DAK | 030-104 | 155 | Placenta: Multifocal transmural ischemic infarction (moderate). Multifocal deciduitis (acute and chronic). Multifocal villitis (acute and chronic, moderate).  Umbilical cord: Squamous metaplasia of the amniotic membrane of the umbilical cord and umbilical insertion. A non-occlusive umbilical thrombus is present.  Fetal heart: focal lymphocytic myocarditis (minimal).  Fetal periadrenal lymph nodes: multifocal erythrophagocytosis (mild) with multifocal neutrophilic lymphadenitis (mild).  Fetal mesenteric lymph node: multifocal neutrophilic lymphadenitis with hemosiderophages (mild).  Fetal axillary lymph node: Edema (mild) with multifocal neutrophlic lymphadenitis and erythrophagocytosis (mild).  Fetal inguinal lymph node: Multifocal erythrophagocytosis (minimal).  Maternal spleen: Multifocal neutrophilic splenitis (minimal) with lymphoid hyperplasia (mild).  Maternal liver: nodular lymphoid hyperplasia (minimal). |
| PBS | 044-105 | 161 | Maternal mesenteric lymph node: lymphoid hyperplasia (moderate).  Decidua parietalis: multifocal decidual necrosis (acute to subacute, moderate). Intralesional vascular fibrinoid necrosis and thrombosis (acute).  Placenta primary disc: Multifocal placenta mineralization.  Placenta secondary disc: Focal extensive infarction of the basal plate (acute to subacute, moderate). Lymphocytic and neutrophilic infiltration (minimal)  Placenta tertiary disc: Diffuse transmural infarction (severe). |
| PBS | 044-106 | 160 | Uterus/placental bed: multifocal lymphocytic and eosinophilic perivascular infiltration of the myometrium (mild).  Decidua - adjacent to myometrium: multifocal lymphocytic perivascular infiltration (mild).  Placenta: Multifocal mineralization of the basal plate and villi (mild to moderate). |
| PBS | 044-107 | 163 | Fetal membranes/decidua parietalis: multifocal acute neutrophilic deciduitis (mild).  Maternal mesenteric lymph nodes: diffuse sinus histiocytes with hemosiderin accumulation (moderate). |
| PBS | 044-108 | 159 | Fetal membranes: congestion and mural hypertrophy of vessels. |
