# Supplementary material for: Fetal loss in pregnant rhesus macaques infected with high-dose African-lineage Zika virus
Source: PLoS Negl Trop Dis. 2022 Aug 4;16(8):e0010623. doi: 10.1371/journal.pntd.0010623 (PMC9380952; doi:10.1371/journal.pntd.0010623)
Supplement: S9 Table — High-dose ZIKV-DAK infants are compared to control infants. (DOCX) [file pntd.0010623.s021.docx]

Table S9. Comparison of neonatal development with the Schneider Neonatal Assessment Protocol (SNAP). High-dose ZIKV-DAK infants are compared to control infants.

| SNAP Construct | Week | Group | Adjusted Means* | Lower 95% CI | Upper 95% CI | Effect size^ | p-value" |
| --- | --- | --- | --- | --- | --- | --- | --- |
| Orientation | 1 | Control | 1.31 | 0.85 | 1.77 | 0.42 | 0.7112 |
|  | 1 | ZIKV-DAK | 1.44 | 0.98 | 1.9 |  |  |
|  | 2 | Control | 1.35 | 0.89 | 1.81 | 0.15 | 0.881 |
|  | 2 | ZIKV-DAK | 1.29 | 0.83 | 1.75 |  |  |
|  | 3 | Control | 1.27 | 0.81 | 1.73 | 0.42 | 0.6016 |
|  | 3 | ZIKV-DAK | 1.45 | 0.99 | 1.91 |  |  |
|  | 4 | Control | 1.58 | 1.12 | 2.04 | 0.84 | 0.3734 |
|  | 4 | ZIKV-DAK | 1.27 | 0.81 | 1.73 |  |  |
| Motor | 1 | Control | 0.7 | 0.34 | 1.06 | 0.55 | 0.4048 |
|  | 1 | ZIKV-DAK | 0.47 | 0.11 | 0.83 |  |  |
|  | 2 | Control | 1.28 | 0.92 | 1.64 | 1.59 | 0.1037 |
|  | 2 | ZIKV-DAK | 0.81 | 0.45 | 1.17 |  |  |
|  | 3 | Control | 1.37 | 1.01 | 1.72 | 0.73 | 0.1836 |
|  | 3 | ZIKV-DAK | 0.99 | 0.63 | 1.35 |  |  |
|  | 4 | Control | 1.58 | 1.22 | 1.94 | 0.48 | 0.3068 |
|  | 4 | ZIKV-DAK | 1.3 | 0.94 | 1.66 |  |  |
| State-Control | 1 | Control | 1.56 | 1.13 | 2 | 0.07 | 0.9579 |
|  | 1 | ZIKV-DAK | 1.58 | 1.14 | 2.02 |  |  |
|  | 2 | Control | 1.12 | 0.69 | 1.56 | 1.12 | 0.2004 |
|  | 2 | ZIKV-DAK | 1.56 | 1.12 | 1.99 |  |  |
|  | 3 | Control | 1.08 | 0.64 | 1.51 | 0.56 | 0.4138 |
|  | 3 | ZIKV-DAK | 1.35 | 0.92 | 1.79 |  |  |
|  | 4 | Control | 0.85 | 0.42 | 1.29 | 0.18 | 0.7603 |
|  | 4 | ZIKV-DAK | 0.95 | 0.52 | 1.39 |  |  |
| Sensory | 1 | Control | 1.25 | 0.97 | 1.53 | 0.32 | 0.36 |
|  | 1 | ZIKV-DAK | 1.44 | 1.16 | 1.72 |  |  |
|  | 2 | Control | 1.32 | 1.05 | 1.6 | 0.62 | 0.9993 |
|  | 2 | ZIKV-DAK | 1.32 | 1.05 | 1.6 |  |  |
|  | 3 | Control | 1.24 | 0.97 | 1.52 | 0.74 | 0.2677 |
|  | 3 | ZIKV-DAK | 1.47 | 1.2 | 1.75 |  |  |
|  | 4 | Control | 1.34 | 1.06 | 1.61 | 0.41 | 0.9263 |
|  | 4 | ZIKV-DAK | 1.35 | 1.08 | 1.63 |  |  |

*adjusted means (adjusted by days before placement with a female, birth weight)

^Cohen’s effect size d (unadjusted)

“p-value for comparing Ctr vs. DAKAR groups
