# Supplementary material for: Fetal loss in pregnant rhesus macaques infected with high-dose African-lineage Zika virus
Source: PLoS Negl Trop Dis. 2022 Aug 4;16(8):e0010623. doi: 10.1371/journal.pntd.0010623 (PMC9380952; doi:10.1371/journal.pntd.0010623)
Supplement: S10 Table — High-dose ZIKV-DAK infants are compared to control infants. (DOCX) [file pntd.0010623.s022.docx]

Table S10. Comparison of gait development with the Nodulus CatWalk. High-dose ZIKV-DAK infants are compared to control infants.

| Group | | | Control infants | | | ZIKV-DAK infants | | | Control vs ZIKV-DAK infants |
| --- | --- | --- | --- | --- | --- | --- | --- | --- | --- |
| Sample size | | | N=2 for week 2; N=3 for week 3&4^ | | | N=3 | | |  |
| **Outcome** | | **Week** | **Mean*** | **Lower 95% CI** | **Upper 95% CI** | **Mean*** | **Lower 95% CI** | **Upper 95% CI** | **p-value** |
| Diagonal Walking Pattern (% of runs) | | 2 | 100 |  |  | 33 |  |  | 0.0476 |
|  | | 3 | 100 |  |  | 56 |  |  | 0.050 |
|  | | 4 | 100 |  |  | 67 |  |  | 0.071 |
| Speed (cm/s) | | 2 | 21.9 | 4.1 | 39.7 | 14.5 | -1.2 | 30.3 | 0.5082 |
|  | | 3 | 35.6 | 19.9 | 51.3 | 17.1 | 1.4 | 32.9 | 0.1115 |
|  | | 4 | 18.6 | 0.4 | 36.9 | 19.6 | 3.8 | 35.3 | 0.936 |
| Duty Cycle (% of time) | Right front limb | 2 | 68.7 | 56.6 | 80.8 | 65.3 | 54 | 76.7 | 0.6494 |
|  |  | 3 | 70.5 | 57.1 | 84 | 67 | 56.1 | 77.9 | 0.6598 |
|  |  | 4 | 68.2 | 55.8 | 80.6 | 66.1 | 55.5 | 76.7 | 0.7857 |
|  | Right hind limb | 2 | 74 | 63.6 | 84.4 | 70.8 | 62.1 | 79.5 | 0.585 |
|  |  | 3 | 71.4 | 61.3 | 81.4 | 70.9 | 62.5 | 79.3 | 0.937 |
|  |  | 4 | 67.3 | 57.9 | 76.7 | 66.5 | 58.2 | 74.8 | 0.8857 |
|  | Left front limb | 2 | 66.7 | 59.5 | 73.9 | 69 | 63 | 75.1 | 0.5736 |
|  |  | 3 | 70.2 | 63.2 | 77.2 | 71.6 | 65.7 | 77.4 | 0.7403 |
|  |  | 4 | 62.4 | 55.8 | 68.9 | 68.8 | 63.1 | 74.6 | 0.1423 |
|  | Left hind limb | 2 | 71.1 | 53.3 | 89 | 79.2 | 62.2 | 96.3 | 0.4868 |
|  |  | 3 | 67.8 | 49.4 | 86.2 | 74.5 | 57.6 | 91.4 | 0.5723 |
|  |  | 4 | 65 | 46.9 | 83.1 | 68.3 | 51.5 | 85.1 | 0.7752 |
| Base of Support (cm) | Front limbs | 2 | 4.7 | 2.9 | 6.6 | 6.1 | 4.5 | 7.7 | 0.2358 |
|  |  | 3 | 3.5 | 1.7 | 5.2 | 5 | 3.4 | 6.6 | 0.1701 |
|  |  | 4 | 4.4 | 2.7 | 6 | 5.4 | 3.9 | 7 | 0.3167 |
|  | Hind limbs | 2 | 6 | 2.7 | 9.2 | 5.4 | 2.3 | 8.5 | 0.7874 |
|  |  | 3 | 4.5 | 1.2 | 7.9 | 5.2 | 2.2 | 8.3 | 0.7399 |
|  |  | 4 | 5.5 | 2.2 | 8.8 | 5 | 2 | 8.1 | 0.8309 |

^*^Adjusted means for duty cycle, base of support: adjusted by number of days before placement with a female, gestational age, birth weight, and speed. Adjusted means for speed: adjusted by number of days before placement with a female, gestational age, birth weight. Adjusted means for diagonal walking pattern: adjusted by the number of days before placement with a female. It was not computationally possible to adjust for gestational age or birth weight.

^Data was missing for two control infants at 14 days and one ZIKV-DAK infant at 14, 21, and 28 days due to their inability to complete the task. Two control animals’ data (1; 21 days and 1; 28 days) was missing due to instrumentation malfunction.
